# Outcomes in patients with acute myeloid leukemia older than 70 years within the last 30 years, a single center experience

**DOI:** 10.1007/s00277-025-06196-2

**Published:** 2025-01-11

**Authors:** Felicitas Schulz, Claudia Roggenbuck, Andrea Kündgen, Annika Kasprzak, Kathrin Nachtkamp, Paul Jäger, Sascha Dietrich, Guido Kobbe, Ulrich Germing, Frank Neumann

**Affiliations:** Department of Hematology, Oncology and Clinical Immunology, Heinrich-Heine University, Moorenstr. 5, Duesseldorf, 40225 Germany

**Keywords:** Acute myeloid leukemia, Prognosis, Treatment strategies

## Abstract

As median age of patients with acute myeloid leukemia is 72 years, older patients continue to be a vulnerable cohort representing significant challenges in clinical practice. Patient-specific comorbidities as well as leukemia-specific unfavorable molecular- and cytogenetics confer even poorer outcomes. Treatment of AML therefore needs to be less toxic to prevent harm while lowering or eradicating leukemic burden to prolong survival. In this retrospective analysis we included 365 older AML patients from the Düsseldorf registry who were diagnosed and treated in our department of hematology over a period of 31 years. Most patients were treated with HMA (37.3%) followed by 35.3% of patients who received either low dose chemotherapy or BSC. 9% of patients were treated with induction chemotherapy while 8.5% of patients received a combination of HMA with venetoclax. 4.1% of patients underwent allografting. At the time of last follow up, 35 patients (9.6%) were still alive. Of those patients who were treated with induction chemotherapy or HMA + venetoclax, 18.2% and 29.0% were still alive, whereas 60% of the patients who underwent allogeneic stem cell transplantation were still alive (*p* < 0.001). Median overall survival of the entire patient population was 6 months. Longest survival was observed in patients who underwent aHSCT with an unreached median overall survival followed by patients who were treated with induction chemotherapy (21 months) or HMA plus venetoclax (11 months). The implementation of HMA + venetoclax and increasing numbers of aHSCT improved prognosis and survival even in older AML patients.

## Introduction

Acute myeloid leukemia (AML) is a heterogeneous disease of older patients with a median age at initial diagnosis of 72 years [1]. The classification of different subtypes according to the World Health Organization (WHO) and International Consensus Classification (ICC) of 2022 is based on cytomorphological, cytogenetical and molecular characteristics. While the 5th edition of the WHO classification still defines AML presenting with a minimum of 20% myeloid blasts in the bone marrow, the ICC enables diagnosing AML with at least 10% myeloid marrow blasts [2, 3]. Compared to the WHO classification of 2016, AML with myelodysplasia-related changes (AML-MRC), the most common subtype in older patients, is now called AML myelodysplasia-related (AML-MR) in WHO 2022 and is split up into AML with myelodysplasia-related gene mutations (AML-MR-M), AML with myelodysplasia-related cytogenetic abnormalities (AML-MR-C) and AML with mutated TP53 [2–4] in ICC 2022.

Although today there are more therapeutic options to treat AML, treatment-related mortality as well as therapy resistance confer a poor prognosis in elderly patients (≥ 70 years) [5, 6]. The proportion of patients with favorable genetic profiles as CBF translocations or isolated NPM1 mutations decreases with increasing age, whereas the number of patients with unfavorable karyotypes and mutations, such as for example TP53, increases [7–9].

Based on the patients’ age and their concomitant comorbidities, a relevant number of patients is not suitable for intensive treatment such as induction therapy or allogeneic hematopoietic stem cell transplantation (aHSCT) while this remains the only curative option for patients suffering from secondary or therapy-related AML [5].

Both the National Comprehensive Cancer Network (NCCN) as well as the European LeukemiaNet (ELN) refrain from defining explicit criteria to decide whether an older patient is eligible for intensive treatment or not [7, 10]. In a considerable proportion of patients, best supportive care often remains the only option.

Several analyses within the last years showed that standard induction therapy in patients older than 75 years of age led to inferior survival and higher early death rates while patients with an ECOG ≥ 3 even had a significantly increased risk of death compared to younger patients [5–7]. However, over the last decades, several therapeutic strategies with different mechanisms of action have emerged. These comprise therapies with hypomethylating agents (HMA) with or without the bcl2-inhibitor venetoclax [11, 12], the addition of gemtuzumab ozogamicin to induction therapy [13, 14], gilteritinib and midostaurin for patients with mutated FLT3 [15, 16], and IDH inhibitors for patients with mutations in IDH1 or IDH2 [17, 18].

In our present analyses, we focus on data from 365 AML patients with a median age of 75 years and a minimum age of 70 years treated at the university hospital in Düsseldorf over a period of more than three decades to describe the impact of different therapies and changes in standard of care.

## Methods

In this retrospective analysis we included 365 older AML patients from the Düsseldorf registry who were diagnosed and treated in our department of hematology over a period of 31 years. Patients were allocated to three different groups depending on time of diagnosis. The periods chosen were before the year 2000, between 2000 and 2017 and later than 2017 because of the rollout of HMAs in 2000 and venetoclax in 2018. Patient characteristics and treatment history were evaluated and survival times according to the various treatment modalities such as non-intensive cytotoxic chemotherapy, induction chemotherapy, allogeneic blood stem cell transplantation (aHSCT), hypomethylating agents (HMA) with or without venetoclax and best supportive care (BSC) including red blood cell and platelet transfusions as well as growth factors were calculated. Patients were classified according to the most intensive treatment they received during the course of the disease. Besides survival, the causes of death, ECOG and Karnofsky index, the ELN risk categories [19] as well as selected molecular genetics were evaluated. Descriptive statistical analyses were performed using the Statistical Package for the Social Sciences (SPSS) version 28 (SPSS, Chicago, IL, USA). Clinical and hematological data at the time of diagnosis were compared using the χ^2^ and Wilcoxon rank sum test. A two-sided p-value of less than 0.05 was considered as statistically significant. The probability of survival was estimated using Kaplan–Meier method [20].

## Results

Patient characteristics at the time of AML diagnosis are shown in Table 1. Median age at diagnosis was 75 years (range 70–93) with 60.5% of patients being male. 68.2% of patients were diagnosed between 2000 and 2017. ECOG performance status at the time of diagnosis was 0 in 9.6% of patients, 1 in 23.0%, 2 in 18.6%, 3 in 9.0% and 4 in 2.2% of patients and remained unknown in 137 patients due to missing data. The majority of patients (57%) were classified as AML-MR while 11.2% of patients suffered from a myeloid neoplasm post cytotoxic therapy as shown in Table 2. Patients were allocated to the different risk groups of ELN 2022 if possible, meaning that they were only classified in case of complete molecular data or enough data to allocate them to the adverse risk group (e.g. complex karyotype or TP53 mutation). The remaining patients were allocated to the ‘undefined’ cohort. A complete molecular panel was only available in 13.3% of patients while for example NPM1 was analyzed in 35% of patients. 6.0% of patients were categorized as favorable according to ELN2022, 5.2% were allocated to the intermediate risk category and 38.6% of patients belonged to the adverse risk group while 50.1% had missing genetic data and could not be classified explicitly. Further details according to molecular genetics as well as cytogenetics at time of diagnosis and the resulting ELN 2022 risk categories can be found in Tables 3 and 4.

Most patients were treated with HMA (37.3%) followed by 35.3% of patients who received either low dose chemotherapy or BSC. 9% of patients were treated with induction chemotherapy while 8.5% of patients received a combination of HMA with venetoclax. 5.8% of patients did not receive any treatment and 4.1% of patients underwent aHSCT as shown in Table 5.

**Fig. 1:**
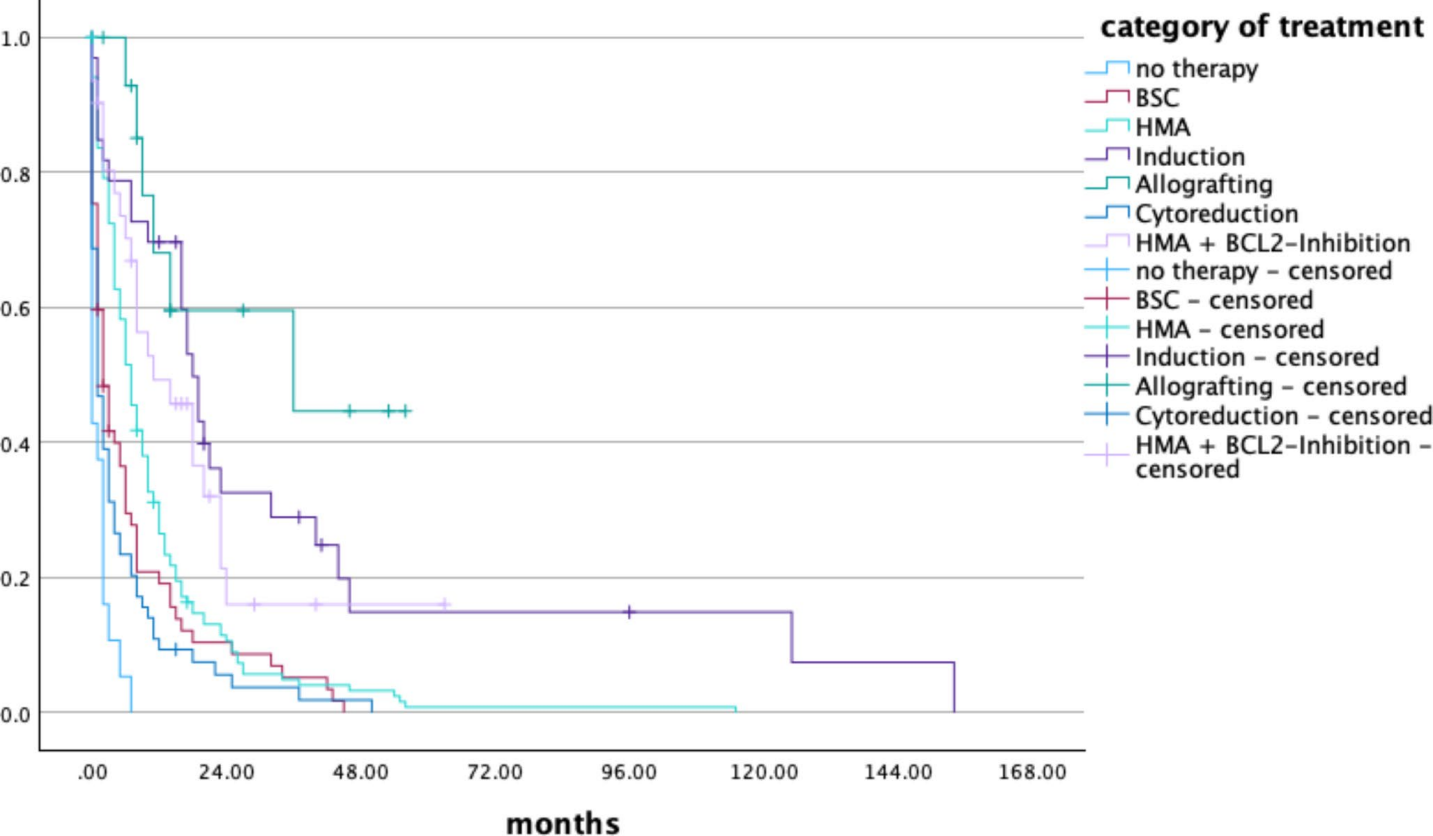
Survival time according to most intensive treatment category

**Fig. 2:**
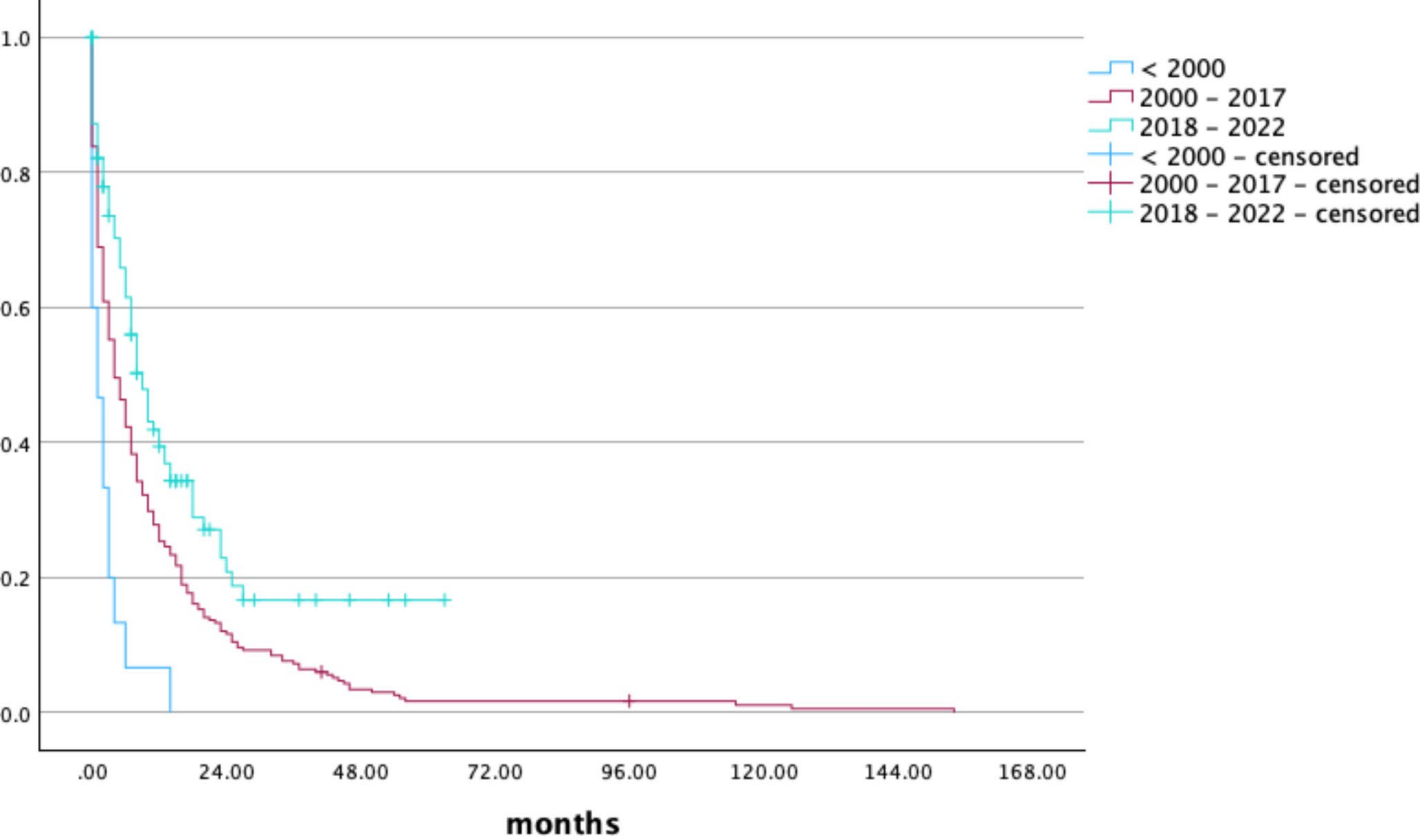
Survival time according to time of diagnosis

Patients who did not receive any therapy as well as those who were treated with low dose chemotherapy alone had a median survival time of 1 month while those ones receiving best supportive care survived 2 months. The use of HMA increased the survival time up to 7 months (*p* < 0.05). A survival time of 11 and 18 months could be observed in patients treated with HMA in combination with venetoclax or induction chemotherapy. Patients who underwent aHSCT had the best prognosis with a median survival time of 36 months as shown in Fig. 1. To further investigate patient’s outcomes, we additionally looked at patients being safely categorized according to ELN 2022 alone and analyzed those 182 patients separately. Patients who received induction chemotherapy survived longer (21 vs. 18 months) while the median overall survival of patients who underwent allogeneic stem cell transplantation was not reached. Detailed information is shown in Table 6. Overall survival of patients according to time of first diagnosis got better with future time of diagnosis and is shown in Fig. 2. Multivariate analysis including patients’ age, gender, ECOG, ELN 2022, time of first diagnosis and type of treatment showed that only the intensity of treatment had indepedent impact on survival, whereas the categorization according to ELN 2022 as well as the other variables did not. Further information regarding 95% CI and p-value are shown in Table 7.

**Table 1:** Patient characteristics at the time of AML diagnosis

		*n* (%)	median (range)
Year of diagnosis	< 2000 2000–2017 > 2017	14 (3.8) 249 (68.2) 102 (28.0)	
Gender	Female	39.5	
	Male	60.5	
Age			75 (70–93)
Medullary blast count (%)			35 (20–99)
Blast count in peripheral blood (%)			28 (0–100)
Hemoglobin g/dl			9.1 (2.1–14.9)
WBC x 1000/µl			5.8 (0.4–365)
ANC x 1000/µl			1.32 (0–113.4)
Platelets x 1000/µl			59 (1–650)
LDH U/l			350 (94–5212)
Fever at diagnosis		34 (9.3)	
Infection at diagnosis		83 (22.7)	
Bleeding at diagnosis		21 (5.8)	
Extramedullary manifestation		14 (3.8)	
ECOG	0	35 (9.6)	
	1	84 (23.0)	
	2	68 (18.6)	
	3	33 (9.0)	
	4	8 (2.2)	
	unknown	137 (37.5)	

**Table 2:** AML subtypes according to WHO 2022

WHO Type	*n* (%)
**AML with recurrent cytogenetics**	**242 (66.3)**
AML MR	208 (57.0)
AML with NPM1	30 (8.2)
AML with CEBPA	3 (0.8)
AML with MECOM-r	1 (0.3)
**AML defined by differentiation**	**77 (21.1)**
AML with minimal differentiation	5 (1.4)
AML without maturation	24 (6.6)
AML with maturation	21 (5.8)
Acute myelomonocytic leukemia	10 (2.7)
Acute monoblastic and monocytic leukemia	16 (4.4)
Pure erythroid leukemia	1 (0.3)
**Myeloid neoplasm post cytotoxic therapy**	**41 (11.2)**
**Unknown**	**5 (1.4)**

**Table 3:** Molecular genetics at time of diagnosis

Type of mutation	*n* (%)
NPM1	30 (8.2)
FLT3 ITD TKD	23 (6.3) 16 (4.4) 7 (1.9)
IDH IDH1 IDH2	22 (6.0) 8 (2.2) 14 (3.8)
ASXL1	18 (4.9)
RUNX1	16 (4.4)
CEBPA	10 (2.7)
TP53	9 (2.5)

**Table 4:** Patients’ risk categories according to ELN 2022

Risk category	*n* (%)
Favorable	22 (6.0)
Intermediate	19 (5.2)
Adverse	141 (38.6)
Undefined	183 (50.1)

**Table 5:** Major characteristics of the different treatment groups

	All patients (*n* = 365)	No treatment (*n* = 21)	BSC (*n* = 65)	Cytoreduction (*n* = 64)	HMA (*n* = 136)	HMA + BCL2 inhibition (*n* = 31)	Induction (*n* = 33)	Allografting (*n* = 15)	*p*-value
Age, median	75	76	78	76	74	76	72	72	
Male	221 (60.5%)	9 (42.9%)	40 (61.5%)	36 (56.3%)	84 (61.8%)	18 (58.1%)	20 (60.6%)	14 (93.3%)	
Year of diagnosis < 2000	15 (4.1%)	6 (28.6%)	7 (10.8%)	2 (3.1%)	0	0	0	0	0.001
Year of diagnosis 2000–2017	248 (67.9%)	14 (66.7%)	44 (67.7%)	54 (84.4%)	103 (75.7%)	0	29 (87.9%)	4 (26.7%)	0.001
Year of diagnosis > 2017	102 (27.9%)	1 (4.8%)	14 (21.5%)	8 (12.5%)	33 (24.3%)	31 (100%)	4 (12.1%)	11 (73.3%)	0.001
Median survival in months (range)	6	1 (0.4–2.2)	2 (0.6–3.4)	1 (0.2–1.8)	7 (5.5–8.5)	11 (1.8–20.2)	18 (14.8–21.2)	36 (21.9–84.3)	0.001

**Table 6:** Major characteristics of the different treatment groups, only patients with exact ELN2022 risk score (*n* = 182)

	All patients (*n* = 182)	No treatment (*n* = 5)	BSC (*n* = 20)	Cytoreduction (*n* = 24)	HMA (*n* = 76)	HMA + BCL2 inhibition (*n* = 30)	Induction (*n* = 14)	Allografting (*n* = 13)	*p*-value
Age, median	74	73	77	77	74	76	72	72	
Male	118 (64.8%)	4 (80%)	14 (70.0%)	15 (62.5%)	47 (61.8%)	17 (56.7%)	9 (64.3%)	12 (92.3%)	
Year of diagnosis < 2000	1 (0.5%)	0	1 (5.0%)	0	0	0	0	0	0.001
Year of diagnosis 2000–2017	98 (53.8%)	5 (100%)	11 (55.0%)	20 (83.3%)	50 (65.8%)	0	10 (71.4%)	2 (15.4%)	0.001
Year of diagnosis > 2017	83 (45.6%)	0	8 (40.0%)	4 (16.7%)	26 (34.2%)	30 (100%)	4 (28.6%)	11 (84.6%)	0.001
Median survival in months (range)	6	1 (0.1–1.2)	1 (0–2.6)	2 (0.7–3.4)	6 (3.7–8.3)	11 (5.1–16.9)	21 (16.5–25.5)	Not reached	0.001

**Table 7:** Significant results of multivariate analysis

Type of treatment	χ^2^	*p*-value	Relative risk	95% CI
• Allografting	48,602	< 0.001		
• Induction	0.689	0.407	1.663	0.5–5.528
• HMA + Venetoclax	4.321	0.038	3.109	1.067–9.058
• HMA	11.875	< 0.001	5.984	2.163–16.555
• No treatment, BSC, cytoreduction	20.807	< 0.001	11.11	3.948–31.265

## Discussion

Acute myeloid leukemia is a disease most frequently diagnosed in older, comorbid patients who are often not eligible for intensive treatment due to pre-existing conditions as well as disease-related problems mostly linked to associated cytopenia. Furthermore, the underlying disease biology and differences in treatment tolerance still lead to poor outcomes. Relying on chronological age alone as a surrogate for patients being eligible for intensive treatment remains a limitation and perpetuates the balancing act between under- and over-treatment resulting in the fact that these patients still comprise a challenge in clinical daily routine.

Until today, there is no consensus regarding optimal therapy and standard of care for older adults with AML [21, 22], which is why we analyzed 365 AML patients with a median age of 75 years treated at our department of hematology over a period of more than three decades. Looking at our cohort, with a minimum age of 70 and the highest age of 93 years, patients were quite old compared to the literature where being categorized as an ‘old patient’ predominantly begins with the age of 60 years [5]. Compared to a large analysis within the United States where between 2000 and 2010 only 40% of patients being newly diagnosed with AML in an age > 60 years received AML-directed therapy [23], the number of patients within our cohort who received no treatment or best supportive care was quite low with only 23.4% between 2000 and 2017. After 2017, 78% of patients were treated with at least an hypomethylating agent being in line with the trend of recent studies towards more frequent use of leukemia-directed therapy in adults aged 65–80 years in the US [24, 25]. With AML-MR being the most frequent and myeloid neoplasm post cytotoxic therapy being the second AML subtype of our cohort, the composition was representative [26]. Since analyses of molecular genetics via next generation sequencing have been further developed and improved over the last 20 years [27], referring data was missing and in our cohort, with NPM1 being the most detected mutation and TP53 mutation only occurring in 2.5% of patients, not representative. Hereby, allocating patients to the different risk categories of ELN 2022, was only possible in 49.9% of cases. Regarding the most intensive treatment option patients did receive, treatment with hypomethylating agents like azacytidine or decitabine was the most frequent option in 37.3% of patients followed by cytoreduction and best supportive care each in a frequency of almost 18%. The median overall survival of 7 months in patients treated with hypomethylating agents was in line with data found in the literature ranging from 7 to 9 months in older AML patients treated with either azacytidine or decitabine [28, 29]. The small number of patients treated with a combination of azacytidine + venetoclax was the result of the approval for treatment with venetoclax in 2021 and its rollout in 2018 and fitted the fact that only patients with date of diagnosis in 2018 or later received this type of therapy. The median survival time of 11 months was shorter than described by DiNardo et al. who observed a median overall survival of 17.5 months in elderly patients [30]. Longer duration of median overall survival with 18 months was seen in our patients undergoing intensive induction chemotherapy which was quite long compared to results of previous studies with a median overall survival < 1 year regarding the well-known 7 + 3 induction regimen as well as CPX-351 [5, 6, 31]. Regarding relapse rates, early mortality or complications like infections or febrile neutropenia, the combination of hypomethylating agents and venetoclax compared to induction therapy turned out to be equivalent or even better [32, 33] being in line with the development within our cohort to treat only a few justified exceptional cases with induction therapy or hypomethylating agents alone instead of a combination of HMA + venetoclax after 2018. Longest median overall survival of 36 months (and even an unreached median overall survival when only looking at the smaller group of 182 patients with a safely known ELN category) could be observed in patients who underwent allografting with only the smallest amount of 4% receiving an allograft but observing increasing numbers with only 4 patients undergoing aHSCT between 2000 und 2017 and 11 patients after 2017. This was again in line with data of the US where the number of aHSCT in older patients has increased visibly in the past decades, rising from less than 0.1% of transplants in 2000 to almost 4% by 2013 [34] and further increasing every year. Expanded knowledge and handling of transplant complications, increasing accessibility to unrelated donors, increased utilization of haploidentical donors and development of reduced-intensity conditioning strategies helped to improve transplant outcome and survival over time while low-intensive induction regimens such as HMA/venetoclax now serve as bridging therapy for remission induction prior to aHSCT making allografting a realistic option even for older patients with AML or other hematologic malignancies. Due to the concept of upfront allogeneic stem cell transplantation in patients not having a high leukemic burden, transplantation rates in our cohort have become quite high with 10% of patients being diagnosed after 2017. Other therapeutic options we were not able to discuss due to missing data were IDH-inhibitors, FLT3-inhibitors, Menin-Inhibitors as well as triplet combinations. In patients with *IDH1* mutation, ivosidenib in combination with HMA improved median overall survival as well as event free survival and response rates compared to monotherapy with HMA [35] while IDH-mutated AML patients who were considered too frail for HMA-based treatment may be offered monotherapy with IDH1/IDH2 inhibitors [17, 36]. The role of FLT3-inhibitors in older patients remains limited as it was mainly combined to intensive induction chemotherapy, but gilteritinib has been approved in the relapsed/refractory setting as monotherapy with a median overall survival of almost ten months [37]. The role of Menin inhibitors in previously untreated, older AML-patients with NPM1 mutations and KMT2A rearrangement is still under investigation in current clinical trials [38] and same applies to triplet combinations like IDH- or FLT3-inhibitors with HMA and venetoclax [39, 40].

Our analyses of 365 older AML patients diagnosed at our department of hematology over a quarter of a century has limitations. Looking at the distribution of patients within our cohort, a relevant number of 68.2% of patients were diagnosed between 2000 and 2017 with only 3.8% of patients being diagnosed before the year 2000 leading to a time bias as well as there is a time-lead bias regarding patients who received an aHSCT due to the fact that patients had to live long enough to experience allogeneic transplantation. Since genetic analyses have evolved over the last 20 years and molecular testing has become more frequent, there is a huge lack of data making important gain of information like ELN classification of the whole cohort impossible. As our analyses are retrospective and documentation of patients has not always been as extensive and disposable as today, we were not able to give evidence about interesting end points like event-free survival, remission or relapse rates as well as treatment-related mortality.

## Conclusion

Older patients suffering from acute myeloid leukemia and hematologic malignancies in general continue to be a vulnerable patient cohort representing significant challenges in clinical daily practice. Patient-specific factors like comorbidities as well as leukemia-specific factors such as underlying unfavorable molecular- and cytogenetics presuppose even poorer outcomes than in younger cohorts. Treatment of AML therefore needs less toxic and more targeted options to prevent harm maintaining quality of life while lowering or eradicating leukemic burden to prolong survival.

As the combination of HMA and venetoclax has enhanced treatment of AML and other therapeutic options in terms of targeted therapies are evolving, the paradigm of conventional 7 + 3 induction is no longer a favored option in vulnerable patient cohorts. With more targeted and simultaneously less toxic therapies, the aim is to widen the landscape of treatment possibilities for elderly patients with AML while prolonging survival and reducing treatment-related mortality.

The combination of upfront allogeneic stem cell transplantation in patients not having a high leukemic burden with less toxic options of conditioning regimens and further experience in transplant complications made allografting a realistic option even for older AML patients.

In conclusion, therapy for older patients with AML has evolved while more therapeutic options are in the pipeline reinforcing even more that care of older and unfit adults needs to essentially stay personalized.

## Data Availability

No datasets were generated or analysed during the current study.

## References

[1] Kraywinkel K, Spix C (2017) Epidemiology of acute leukemia in Germany. Onkologe 23:499–503

[2] Khoury JD, Solary E, Abla O, Akkari Y, Alaggio R, Apperley JF, Bejar R, Berti E, Busque L, Chan JKC, Chen W, Chen X, Chng WJ, Choi JK, Colmenero I, Coupland SE, Cross NCP, De Jong D, Elghetany MT, Takahashi E, Emile JF, Ferry J, Fogelstrand L, Fontenay M, Germing U, Gujral S, Haferlach T, Harrison C, Hodge JC, Hu S, Jansen JH, Kanagal-Shamanna R, Kantarjian HM, Kratz CP, Li XQ, Lim MS, Loeb K, Loghavi S, Marcogliese A, Meshinchi S, Michaels P, Naresh KN, Natkunam Y, Nejati R, Ott G, Padron E, Patel KP, Patkar N, Picarsic J, Platzbecker U, Roberts I, Schuh A, Sewell W, Siebert R, Tembhare P, Tyner J, Verstovsek S, Wang W, Wood B, Xiao W, Yeung C, Hochhaus A (2022) The 5th edition of the World Health Organization Classification of Haematolymphoid Tumours: myeloid and Histiocytic/Dendritic neoplasms. Leukemia 36(7):1703–1719. 10.1038/s41375-022-01613-1 35732831 10.1038/s41375-022-01613-1 PMC9252913

[3] Daniel A, Arber A, Orazi RP, Hasserjian MJ, Borowitz KR, Calvo H-M, Kvasnicka SA, Wang A, Bagg T, Barbui S, Branford CE, Bueso-Ramos JE, Cortes PD, Cin, Courtney D, DiNardo EJ, Duncavage BL, Ebert, Elihu H, Estey F, Facchetti K, Foucar N, Gangat U, Gianelli LA, Godley S, Hobbs R, Hoffman EJ, Jabbour J-J, Kiladjian RA, Larson, Michelle M, Le Beau ML-C, Loh B, Löwenberg E, Macintyre L, Malcovati CG, Mullighan OM, Odenike S, Ogawa A, Orfao E, Papaemmanuil F, Passamonti K, Porkka C-H, Pui JP Radich, Andreas Reiter, Maria Rozman, Martina Rudelius, Savona MR, Schiffer CA, Annette Schmitt-Graeff, Akiko Shimamura, Jorge Sierra, Stock WA, Stone RM, Vannucchi MS, Vyas P, Wei AH (2022) Olga K. Weinberg, Agnieszka Wierzbowska, Mario Cazzola, Hartmut Döhner, Ayalew Tefferi; International Consensus Classification of Myeloid Neoplasms and Acute Leukemias: integrating morphologic, clinical, and genomic data. *Blood*; 140 (11): 1200–1228. 10.1182/blood.2022015850 10.1182/blood.2022015850 PMC9479031 35767897

[4] Daniel A, Arber A, Orazi R, Hasserjian Jürgen, Thiele MJ, Borowitz, Michelle M, Le Beau CD, Bloomfield M, Cazzola JW, Vardiman (2016) The 2016 revision to the World Health Organization classification of myeloid neoplasms and acute leukemia. Blood 127(20):2391–2405. 10.1182/blood-2016-03-643544 27069254 10.1182/blood-2016-03-643544

[5] Appelbaum FR, Gundacker H, Head DR, Slovak ML, Willman CL, Godwin JE, Anderson JE, Petersdorf SH (2006) Age and acute myeloid leukemia. Blood 107(9):3481–3485. 10.1182/blood-2005-09-3724 16455952 10.1182/blood-2005-09-3724 PMC1895766

[6] Kantarjian H, O’brien S, Cortes J, Giles F, Faderl S, Jabbour E, Garcia-Manero G, Wierda W, Pierce S, Shan J, Estey E (2006) Results of intensive chemotherapy in 998 patients age 65 years or older with acute myeloid leukemia or high-risk myelodysplastic syndrome: predictive prognostic models for outcome. Cancer 106(5):1090–1098. 10.1002/cncr.21723 16435386 10.1002/cncr.21723

[7] O’Donnell MR, Tallman MS, Abboud CN, Altman JK, Appelbaum FR, Arber DA, Bhatt V, Bixby D, Blum W, Coutre SE, De Lima M, Fathi AT, Fiorella M, Foran JM, Gore SD, Hall AC, Kropf P, Lancet J, Maness LJ, Marcucci G, Martin MG, Moore JO, Olin R, Peker D, Pollyea DA, Pratz K, Ravandi F, Shami PJ, Stone RM, Strickland SA, Wang ES, Wieduwilt M, Gregory K, Ogba N (2017) Acute myeloid leukemia, Version 3.2017, NCCN Clinical Practice guidelines in Oncology. J Natl Compr Canc Netw 15(7):926–957. 10.6004/jnccn.2017.0116 28687581 10.6004/jnccn.2017.0116

[8] Pollyea DA, Kohrt HE, Medeiros BC (2011) Acute myeloid leukaemia in the elderly: a review. Br J Haematol 152(5):524–542. 10.1111/j.1365-2141.2010.08470.x 21314823 10.1111/j.1365-2141.2010.08470.x

[9] Dombret H, Raffoux E, Gardin C (2008) Acute myeloid leukemia in the elderly. Semin Oncol 35(4):430–438. 10.1053/j.seminoncol.2008.04.013 18692693 10.1053/j.seminoncol.2008.04.013

[10] Döhner H, Andrew H, Wei FR, Appelbaum C, Craddock CD, DiNardo, Hervé Dombret BL, Ebert P, Fenaux LA, Godley RP, Hasserjian RA, Larson RL, Levine J, Sierra EM, Stein MS, Tallman (2022) Hwei-Fang Tien, Jianxiang Wang, Agnieszka Wierzbowska, Bob Löwenberg; Diagnosis and management of AML in adults: 2022 recommendations from an international expert panel on behalf of the ELN. *Blood*; 140 (12): 1345–1377. 10.1182/blood.2022016867 10.1182/blood.2022016867 35797463

[11] DiNardo CD, Pratz KW, Letai A, Jonas BA, Wei AH, Thirman M, Arellano M, Frattini MG, Kantarjian H, Popovic R, Chyla B, Xu T, Dunbar M, Agarwal SK, Humerickhouse R, Mabry M, Potluri J, Konopleva M, Pollyea DA (2018) Safety and preliminary efficacy of venetoclax with decitabine or azacitidine in elderly patients with previously untreated acute myeloid leukaemia: a non-randomised, open-label, phase 1b study. Lancet Oncol 19(2):216–228. 10.1016/S1470-2045(18)30010-X 29339097 10.1016/S1470-2045(18)30010-X

[12] DiNardo CD, Jonas BA, Pullarkat V, Thirman MJ, Garcia JS, Wei AH, Konopleva M, Döhner H, Letai A, Fenaux P, Koller E, Havelange V, Leber B, Esteve J, Wang J, Pejsa V, Hájek R, Porkka K, Illés Á, Lavie D, Lemoli RM, Yamamoto K, Yoon SS, Jang JH, Yeh SP, Turgut M, Hong WJ, Zhou Y, Potluri J, Pratz KW (2020) Azacitidine and Venetoclax in previously untreated Acute Myeloid Leukemia. N Engl J Med 383(7):617–629. 10.1056/NEJMoa2012971 32786187 10.1056/NEJMoa2012971

[13] Hills RK, Castaigne S, Appelbaum FR, Delaunay J, Petersdorf S, Othus M, Estey EH, Dombret H, Chevret S, Ifrah N, Cahn JY, Récher C, Chilton L, Moorman AV, Burnett AK (2014) Addition of gemtuzumab ozogamicin to induction chemotherapy in adult patients with acute myeloid leukaemia: a meta-analysis of individual patient data from randomised controlled trials. Lancet Oncol 15(9):986–996. 10.1016/S1470-2045(14)70281-5 25008258 10.1016/S1470-2045(14)70281-5 PMC4137593

[14] Lambert J, Pautas C, Terré C, Raffoux E, Turlure P, Caillot D, Legrand O, Thomas X, Gardin C, Gogat-Marchant K, Rubin SD, Benner RJ, Bousset P, Preudhomme C, Chevret S, Dombret H, Castaigne S (2019) Gemtuzumab ozogamicin for *de novo* acute myeloid leukemia: final efficacy and safety updates from the open-label, phase III ALFA-0701 trial. Haematologica 104(1):113–119. 10.3324/haematol.2018.188888 30076173 10.3324/haematol.2018.188888 PMC6312010

[15] Stone RM, Mandrekar SJ, Sanford BL, Laumann K, Geyer S, Bloomfield CD, Thiede C, Prior TW, Döhner K, Marcucci G, Lo-Coco F, Klisovic RB, Wei A, Sierra J, Sanz MA, Brandwein JM, de Witte T, Niederwieser D, Appelbaum FR, Medeiros BC, Tallman MS, Krauter J, Schlenk RF, Ganser A, Serve H, Ehninger G, Amadori S, Larson RA, Döhner H (2017) Midostaurin plus chemotherapy for Acute myeloid leukemia with a FLT3 mutation. N Engl J Med 377(5):454–464. 10.1056/NEJMoa1614359 28644114 10.1056/NEJMoa1614359 PMC5754190

[16] Perl AE, Altman JK, Cortes J, Smith C, Litzow M, Baer MR, Claxton D, Erba HP, Gill S, Goldberg S, Jurcic JG, Larson RA, Liu C, Ritchie E, Schiller G, Spira AI, Strickland SA, Tibes R, Ustun C, Wang ES, Stuart R, Röllig C, Neubauer A, Martinelli G, Bahceci E, Levis M (2017) Selective inhibition of FLT3 by gilteritinib in relapsed or refractory acute myeloid leukaemia: a multicentre, first-in-human, open-label, phase 1–2 study. Lancet Oncol 18(8):1061–1075. 10.1016/S1470-2045(17)30416-3 28645776 10.1016/S1470-2045(17)30416-3 PMC5572576

[17] Stein EM, DiNardo CD, Pollyea DA, Fathi AT, Roboz GJ, Altman JK, Stone RM, DeAngelo DJ, Levine RL, Flinn IW, Kantarjian HM, Collins R, Patel MR, Frankel AE, Stein A, Sekeres MA, Swords RT, Medeiros BC, Willekens C, Vyas P, Tosolini A, Xu Q, Knight RD, Yen KE, Agresta S, de Botton S, Tallman MS (2017) Enasidenib in mutant *IDH2* relapsed or refractory acute myeloid leukemia. Blood 130(6):722–731. 10.1182/blood-2017-04-779405 28588020 10.1182/blood-2017-04-779405 PMC5572791

[18] DiNardo CD, Stein AS, Stein EM, Fathi AT, Frankfurt O, Schuh AC, Döhner H, Martinelli G, Patel PA, Raffoux E, Tan P, Zeidan AM, de Botton S, Kantarjian HM, Stone RM, Frattini MG, Lersch F, Gong J, Gianolio DA, Zhang V, Franovic A, Fan B, Goldwasser M, Daigle S, Choe S, Wu B, Winkler T, Vyas P (2021) Mutant isocitrate dehydrogenase 1 inhibitor Ivosidenib in Combination with azacitidine for newly diagnosed Acute myeloid leukemia. J Clin Oncol 39(1):57–65. 10.1200/JCO.20.01632 33119479 10.1200/JCO.20.01632 PMC7771719

[19] Döhner H, Wei AH, Appelbaum FR, Craddock C, DiNardo CD, Dombret H, Ebert BL, Fenaux P, Godley LA, Hasserjian RP, Larson RA, Levine RL, Miyazaki Y, Niederwieser D, Ossenkoppele G, Röllig C, Sierra J, Stein EM, Tallman MS, Tien HF, Wang J, Wierzbowska A, Löwenberg B (2022) Diagnosis and management of AML in adults: 2022 recommendations from an international expert panel on behalf of the ELN. Blood 140(12):1345–1377. 10.1182/blood.2022016867 35797463 10.1182/blood.2022016867

[20] Kaplan EL, Meier P (1958) Nonparametric estimation from incomplete observations. J Am Stat Assoc 53:457–481. 10.2307/2281868

[21] Klepin HD (2016) Myelodysplastic syndromes and Acute Myeloid Leukemia in the Elderly. Clin Geriatr Med 32(1):155–173. 10.1016/j.cger.2015.08.010 26614866 10.1016/j.cger.2015.08.010 PMC4664149

[22] Sekeres MA, Guyatt G, Abel G, Alibhai S, Altman JK, Buckstein R, Choe H, Desai P, Erba H, Hourigan CS, LeBlanc TW, Litzow M, MacEachern J, Michaelis LC, Mukherjee S, O’Dwyer K, Rosko A, Stone R, Agarwal A, Colunga-Lozano LE, Chang Y, Hao Q, Brignardello-Petersen R (2020) American Society of Hematology 2020 guidelines for treating newly diagnosed acute myeloid leukemia in older adults. Blood Adv 4(15):3528–3549. 10.1182/bloodadvances.2020001920 32761235 10.1182/bloodadvances.2020001920 PMC7422124

[23] PDF Version - SEER Cancer Statistics Review (CSR) (2016) 1975– SEER Accessed January 18, 2020. https://seer.cancer.gov/csr/1975_2016/sections.html

[24] Juliusson G, Antunovic P, Derolf A et al (2009) Age and acute myeloid leukemia: real world data on decision to treat and outcomes from the Swedish Acute Leukemia Registry. Blood 113(18):4179–4187. 10.1182/blood-2008-07-172007 19008455 10.1182/blood-2008-07-172007

[25] Löwenberg B, Zittoun R, Kerkhofs H et al (1989) On the value of intensive remission-induction chemotherapy in elderly patients of 65 + years with acute myeloid leukemia: a randomized phase III study of the European Organization for Research and Treatment of Cancer Leukemia Group. J Clin Oncol off J Am Soc Clin Oncol 7(9):1268–1274. 10.1200/JCO.1989.7.9.1268 10.1200/JCO.1989.7.9.1268 2475589

[26] Büchner T, Berdel WE, Haferlach C, Haferlach T, Schnittger S, Müller-Tidow C, Braess J, Spiekermann K, Kienast J, Staib P, Grüneisen A, Kern W, Reichle A, Maschmeyer G, Aul C, Lengfelder E, Sauerland MC, Heinecke A, Wörmann B, Hiddemann W (2009) Age-related risk profile and chemotherapy dose response in acute myeloid leukemia: a study by the German Acute Myeloid Leukemia Cooperative Group. J Clin Oncol 27(1):61–69. 10.1200/JCO.2007.15.4245 19047294 10.1200/JCO.2007.15.4245

[27] Haferlach T (2020) Advancing leukemia diagnostics: role of Next Generation sequencing (NGS) in acute myeloid leukemia. Hematol Rep 12(Suppl 1):8957. 10.4081/hr.2020.8957 33042506 10.4081/hr.2020.8957 PMC7520852

[28] Récher C, Röllig C, Bérard E, Bertoli S, Dumas PY, Tavitian S, Kramer M, Serve H, Bornhäuser M, Platzbecker U, Müller-Tidow C, Baldus CD, Martínez-Cuadrón D, Serrano J, Martínez-Sánchez P, Arbolí ER, Gil C, Bergua J, Bernal T, de la Fuente Burguera A, Delabesse E, Bidet A, Pigneux A, Montesinos P (2022) Long-term survival after intensive chemotherapy or hypomethylating agents in AML patients aged 70 years and older: a large patient data set study from European registries. Leukemia 36(4):913–922. 10.1038/s41375-021-01425-9 34775483 10.1038/s41375-021-01425-9 PMC8979811

[29] Zeidan AM, Wang R, Wang X, Shallis RM, Podoltsev NA, Bewersdorf JP, Huntington SF, Neparidze N, Giri S, Gore SD, Davidoff AJ, Ma X (2020) Clinical outcomes of older patients with AML receiving hypomethylating agents: a large population-based study in the United States. Blood Adv 4(10):2192–2201. 10.1182/bloodadvances.2020001779 32433746 10.1182/bloodadvances.2020001779 PMC7252544

[30] DiNardo CD, Pratz K, Pullarkat V, Jonas BA, Arellano M, Becker PS, Frankfurt O, Konopleva M, Wei AH, Kantarjian HM, Xu T, Hong WJ, Chyla B, Potluri J, Pollyea DA, Letai A (2019) Venetoclax combined with decitabine or azacitidine in treatment-naive, elderly patients with acute myeloid leukemia. Blood 133(1):7–17. 10.1182/blood-2018-08-868752 30361262 10.1182/blood-2018-08-868752 PMC6318429

[31] Lancet JE, Uy GL, Newell LF, Lin TL, Ritchie EK, Stuart RK, Strickland SA, Hogge D, Solomon SR, Bixby DL, Kolitz JE, Schiller GJ, Wieduwilt MJ, Ryan DH, Faderl S, Cortes JE (2021) CPX-351 versus 7 + 3 cytarabine and daunorubicin chemotherapy in older adults with newly diagnosed high-risk or secondary acute myeloid leukaemia: 5-year results of a randomised, open-label, multicentre, phase 3 trial. Lancet Haematol 8(7):e481–e491. 10.1016/S2352-3026(21)00134-4 34171279 10.1016/S2352-3026(21)00134-4

[32] Maiti A, Qiao W, Sasaki K, Ravandi F, Kadia TM, Jabbour EJ, Daver NG, Borthakur G, Garcia-Manero G, Pierce SA, Montalbano KS, Pemmaraju N, Naqvi K, Ohanian M, Short NJ, Alvarado Y, Takahashi K, Yilmaz M, Jain N, Kornblau SM, Andreeff M, Bose P, Ferrajoli A, Issa GC, Masarova L, Thompson PA, Rausch CR, Ning J, Kantarjian HM, DiNardo CD, Konopleva MY (2021) Venetoclax with decitabine vs intensive chemotherapy in acute myeloid leukemia: a propensity score matched analysis stratified by risk of treatment-related mortality. Am J Hematol 96(3):282–291. 10.1002/ajh.26061 33264443 10.1002/ajh.26061 PMC8128145

[33] Matthews AH, Perl AE, Luger SM, Loren AW, Gill SI, Porter DL, Babushok DV, Maillard IP, Carroll MP, Frey NV, Hexner EO, Martin ME, McCurdy SR, Stadtmauer EA, Paralkar VR, Bruno XJ, Hwang WT, Margolis D, Pratz KW (2022) Real-world effectiveness of CPX-351 vs venetoclax and azacitidine in acute myeloid leukemia. Blood Adv 6(13):3997–4005. 10.1182/bloodadvances.2022007265 35507945 10.1182/bloodadvances.2022007265 PMC9278286

[34] Muffly L, Pasquini MC, Martens M, Brazauskas R, Zhu X, Adekola K, Aljurf M, Ballen KK, Bajel A, Baron F, Battiwalla M, Beitinjaneh A, Cahn JY, Carabasi M, Chen YB, Chhabra S, Ciurea S, Copelan E, D’Souza A, Edwards J, Foran J, Freytes CO, Fung HC, Gale RP, Giralt S, Hashmi SK, Hildebrandt GC, Ho V, Jakubowski A, Lazarus H, Luskin MR, Martino R, Maziarz R, McCarthy P, Nishihori T, Olin R, Olsson RF, Pawarode A, Peres E, Rezvani AR, Rizzieri D, Savani BN, Schouten HC, Sabloff M, Seftel M, Seo S, Sorror ML, Szer J, Wirk BM, Wood WA, Artz A (2017) Increasing use of allogeneic hematopoietic cell transplantation in patients aged 70 years and older in the United States. Blood 130(9):1156–1164. 10.1182/blood-2017-03-772368 28674027 10.1182/blood-2017-03-772368 PMC5580273

[35] Montesinos P, Recher C, Vives S, Zarzycka E, Wang J, Bertani G, Heuser M, Calado RT, Schuh AC, Yeh SP, Daigle SR, Hui J, Pandya SS, Gianolio DA, de Botton S, Döhner H (2022) Ivosidenib and Azacitidine in IDH1-Mutated Acute myeloid leukemia. N Engl J Med 386(16):1519–1531. 10.1056/NEJMoa2117344 35443108 10.1056/NEJMoa2117344

[36] Roboz, G. J., DiNardo, C. D., Stein, E. M., de Botton, S., Mims, A. S., Prince, G.T.,… Stone, R. M. (2020). Ivosidenib induces deep durable remissions in patients with newly diagnosed IDH1-mutant acute myeloid leukemia.Blood, The Journal of the American Society of Hematology, 135(7), 463–471 10.1182/blood.2019002140 PMC7019193 31841594

[37] Perl AE, Martinelli G, Cortes JE, Neubauer A, Berman E, Paolini S, Montesinos P, Baer MR, Larson RA, Ustun C, Fabbiano F, Erba HP, Di Stasi A, Stuart R, Olin R, Kasner M, Ciceri F, Chou WC, Podoltsev N, Recher C, Yokoyama H, Hosono N, Yoon SS, Lee JH, Pardee T, Fathi AT, Liu C, Hasabou N, Liu X, Bahceci E, Levis MJ (2019) Gilteritinib or Chemotherapy for relapsed or refractory FLT3-Mutated AML. N Engl J Med 381(18):1728–1740. 10.1056/NEJMoa1902688 31665578 10.1056/NEJMoa1902688

[38] Issa, G. C., Aldoss, I., DiPersio, J. F., Cuglievan, B., Stone, R. M., Arellano, M.L.,… Stein, E. (2022). The menin inhibitor SNDX-5613 (revumenib) leads to durable responses in patients (Pts) with KMT2A-rearranged or NPM1 mutant AML: updated results of a phase (Ph) 1 study.Blood, 140(Supplement 1), 150–152

[39] Maiti A, DiNardo CD, Daver NG, Rausch CR, Ravandi F, Kadia TM, Pemmaraju N, Borthakur G, Bose P, Issa GC, Short NJ, Yilmaz M, Montalban-Bravo G, Ferrajoli A, Jabbour EJ, Jain N, Ohanian M, Takahashi K, Thompson PA, Loghavi S, Montalbano KS, Pierce S, Wierda WG, Kantarjian HM, Konopleva MY (2021) Triplet therapy with venetoclax, FLT3 inhibitor and decitabine for FLT3-mutated acute myeloid leukemia. Blood Cancer J 11(2):25. 10.1038/s41408-021-00410-w 33563904 10.1038/s41408-021-00410-w PMC7873265

[40] Lachowiez, C. A., Borthakur, G., Loghavi, S., Zeng, Z., Kadia, T. M., Masarova, L.,… Dinardo, C. D. (2021). A phase Ib/II study of ivosidenib with venetoclax+/-azacitidine in IDH1-mutated myeloid malignancies. 10.1158/2643-3230.BCD-22-0205 PMC10320628 37102976

